# Complete genome sequence of *Pediococcus pentosaceus* 5.5 8-1 isolated from a pasture-kept Holstein Friesian cattle

**DOI:** 10.1128/mra.00849-25

**Published:** 2025-11-12

**Authors:** Fritz Titgemeyer, Nadine Mariani Corea, Frank Meyer, Sebastian W. Fischer

**Affiliations:** 1 Department of Food - Nutrition - Facilities, FH Münster 39002, Münster, Germany; 2 Institute for Hygiene and Public Health, University Clinics Bonn, Bonn, Germany; University of Pittsburgh School of Medicine, Pittsburgh, Pennsylvania, USA

**Keywords:** lactic acid bacteria, probiotics, protective cultures, nanopore, postbiotics, food safety, food fermentation

## Abstract

*A Pediococcus pentosaceus* strain was isolated from a Holstein Friesian milk cow on an organic farm. The complete genome sequence of 1.73 Mbp and 1,671 predicted genes of *P. pentosaceus* 5.5 8-1 (5_5_8_1 at NCBI databases) was determined for further investigation of environmental biodiversity and application in food fermentation.

## ANNOUNCEMENT

*Pediococci* are lactic acid bacteria used for food fermentation and food preservation (1–3). They exert numerous probiotic effects and produce small beneficial molecules, so-called postbiotics (3–6). Strain 05.5 8-1 was isolated on an organic dairy farm from foremilk from one udder quarter of a Holstein Friesian cow. Foremilk was diluted in NaCl–Tween solution (9 g/L NaCl; 20 ml/L Tween 80). Dilutions were plated onto de Man, Rogosa, and Sharpe (MRS)-Bouillon-agar plates (0.5 g/L cysteine; 10 mg/L bromophenol blue) and incubated in an anaerobic jar (30°C; 48 h) (7). DNA from single colonies was subjected to polymerase chain reactions of the *rrnA* gene encoding 16S rRNA with primers 27f (5′-AGAGTTTGATCCTGGCTCAG-3′) and N1492r (5′-TACGGYTACCTTGTTAYGACTT-3′) (8). The Sanger sequenced DNA of a 1,067 bp was identical to *rrnA* of *Pediococcus pentosaceus* ATCC 25745 (NC_008525.1).

The strain was cultured on MRS agar with 0.5 g/L cysteine for 48 h at 30°C. Genomic DNA was isolated using the Wizard HMW DNA Extraction Kit (Promega, USA) and quantified with a DeNovix QFX fluorometer (DeNovix, USA). A rapid sequencing library was prepared with the Oxford Nanopore SQK-RAD004 kit and loaded (30 µL) onto a Flongle Flow Cell R9.4.1 (FLO-FLG001) mounted on a MinION Mk1B device running MinKNOW v22.08.9 (9). Raw reads for assembling were basecalled with Guppy v6.3.8 (high-accuracy model dna_r9.4.1_450bps_hac.cfg) and re-basecalled for additional polishing steps with Dorado v0.9.6 (model dna_r9.4.1_e8_sup@v3.6 with foundation) (10, 11). Reads with a mean Phred quality ≥Q9 were retained. Adapter trimming was performed with Porechop v0.2.4 (12). Reads were filtered (Filtlong v0.2.1) to discard the worst 10% by quality and all reads <1 kb (13). The final data set comprised 20,519 reads (71.62 Mbp; read N50 = 4,949 bp; median/mean read length = 2,236/3,490 bp; longest read = 140,560 bp; mean read Q = 11; 43.6 %/10.7% of bases reached ≥Q20/≥Q30, respectively). Quality metrics were obtained with Nanoq v0.1.0 and SeqKit v2.9.0 (14, 15). Assembling was done by Trycycler v0.5.4. Sub‑assemblies were generated with Flye v2.9.2, Raven v1.8.2, and Miniasm v0.3 (16–20). The draft consensus was polished with Medaka v1.8.0. Circularity and correctness were verified with Bandage v0.9.0 (21). For additional polishing, re-basecalled reads were aligned via Dorado Aligner against the assembly and filtered (Samtools v1.22.1; MAPQ ≥20; flags QC-failed 0x200, secondary 0x100, and supplementary 0x800 excluded); ≥1 kb) (11, 22). One Medaka v2.1.0 round and two Homopolish v0.4.1 rounds (polish, modpolish) were then applied. The final circular chromosome was oriented to *dnaA* as the starting point (Dnaapler v1.2.0) (23–25).

FastANI and QUAST v5.3.0, using *P. pentosaceus* ATCC 25745 (NC_008525.1) as reference, yielded the metrics in Table 1; BUSCO v5.8.3 (data set *pediococcus_odb12*) reported 98.9% completeness (26–28). CheckM2 (specific neural-network model) confirmed 99.98% completeness and 0.13% contamination (29). Read depth, obtained by remapping the filtered reads with minimap2 v2.30 and samtools v1.22.1, showed a mean coverage of 27× across the chromosome (22, 30, 31). Genome features were predicted with Bakta v1.8.2 (database v5.0 light), identifying 1,671 protein-coding sequences, 15 rRNA genes, 56 tRNA genes, 1 tmRNA, and 3 ncRNAs (total features = 1,769) (32).

**TABLE 1:** Assembly and quality metrics for the complete chromosome of *P. pentosaceus* 5.5 8-1

Replicon	Length (bp)	GC (%)	BUSCO completeness (%)	CheckM2 completeness/contamination (%)	Genome fraction (%)	Average nucleotide identity
Chromosome	1,731,209	37.4	98.9	99.98/0.13	86.66	98.61

## Data Availability

The whole-genome sequencing data for Pediococcus pentosaceus isolate 5.5 8-1 (5_5_8_1) have been deposited in the NCBI databases under BioSample (SAMN50255360) in BioProject (PRJNA1297324). The raw sequencing reads are available under SRA entries (SRX29883225) and (SRX29883226). The assembled and annotated genome is available under GenBank accession numbers (CP197206) and (GCF_052059785.1).
